# Observation of the Intervention Effect of Biofeedback Therapy Combined With Cluster Nursing on Perioperative Constipation in Patients With Thoracolumbar Fracture

**DOI:** 10.3389/fsurg.2022.847068

**Published:** 2022-03-07

**Authors:** Jin Luo, Nan Xie, Liping Yang

**Affiliations:** ^1^Department of Spine Surgery, Suining Central Hospital, Suining, China; ^2^Nursing Department, Suining Central Hospital, Suining, China; ^3^Department of Arthrosurgery, Zhuji People's Hospital of Zhejiang Province, Zhuji, China

**Keywords:** thoracolumbar fracture, constipation, perioperative, biofeedback therapy, cluster nursing

## Abstract

**Purpose:**

To discuss the intervention effect of biofeedback therapy combined with cluster nursing on perioperative constipation in patients with thoracolumbar fracture.

**Methods:**

From June 2019 to June 2020, a total of 482 patients with thoracolumbar fracture who were treated by surgery in our department were selected. The random number table method was used to divide into experimental group (*n* = 241) and control group (*n* = 241). The control group was given routine constipation care, the experimental group was given biofeedback therapy combined with cluster nursing based on the control group. The constipation score, Bristol stool scale score, the short health questionnaire (SF-36) scale score, and the satisfaction of two groups were observed.

**Results:**

The constipation scores of the experimental group were lower than those of the control group, while the Bristol stool scale score, SF-36 score, and satisfaction degree of the experimental group were higher than those of the control group (*p* < 0.05).

**Conclusion:**

Biofeedback therapy combined with cluster nursing has a good intervention effect in perioperative constipation of patients with thoracolumbar fracture, which can reduce the degree of constipation, improve stool traits, improve the quality of life, and improve the satisfaction of patients.

## Introduction

Thoracolumbar fracture is a kind of spinal injury disease in the transition region from thoracic segment to lumbar segment, this area is prone to stress concentration, which can lead to fracture (1). Thoracolumbar fracture accounts for >60% of spinal injuries. When the body suffers from acute injuries, such as car accidents, falling from a height, or when the elderly have risk factors, such as osteoporosis, it can lead to thoracolumbar fracture (2, 3). Surgical intervention is the most important treatment for the thoracolumbar fracture. However, due to the influence of long-term bed rest and psychological factors, thoracolumbar fracture patients are prone to gastrointestinal dysfunction during the perioperative period, among which constipation is more common (4). The main clinical manifestations of constipation are strenuous defecation, reduced defecation times, incomplete defecation or dry, and hard stool (5). The incidence of constipation can occur in 50–90% of patients with thoracolumbar fractures, which usually occur within 24 h after fracture and sometimes lasts for more than 7 d (6). Constipation will weaken the gastrointestinal barrier ability of patients, increase the incidence of anorectal diseases, increase hypertension, increase oxygen consumption, and even induce cardiovascular and cerebrovascular diseases in forced defecation. Constipation can also lead to the poor nutritional status of patients, causing patients to have problems, such as irritability and anxiety. In severe cases, it may even affect fracture healing, prolong hospitalization time, and reduce patients' quality of life (7–9). Therefore, perioperative constipation in patients with thoracolumbar fracture requires attention and appropriate treatment.

Biofeedback therapy, with the help of modern physiological scientific instruments, records much imperceptible information of people's psychological and physiological processes, such as electromyography, skin electricity, electroencephalography, then amplifies and converts them into information that people can understand, which can be displayed in the form of vision and hearing with easy recognition (10). When individuals are aware of these physiological or pathological changes, medical personnel train people to consciously control the changes in signal activities and regulate abnormal physiological responses, so as to improve physical functions, which have the effect of disease prevention and disease treatment (11).

Cluster nursing based on evidence-based medicine, according to the principle of “people-oriented” and combined with the actual situation of the hospital and patients, to provide patients with a variety of high-quality nursing services, which can improve the overall quality of nursing, is conducive to improving the prognosis of patients (12). The cluster nursing intervention program is not only the implementation process of nursing methods but also the management process of nursing quality. At the same time, it also reminds medical staff to pay attention to the relationship between a certain measure and prognosis, thus improving the clinical treatment effect (13). A clustering strategy is a set of intervention measures related to a certain disease process, and compared with a single implementation, the intervention effect is better.

In this study, the intervention effect of biofeedback therapy combined with cluster nursing on perioperative constipation in patients with thoracolumbar fracture was observed, in order to improve the living standard of patients.

## Materials and Methods

### Research Object

From June 2019 to June 2020, a total of 482 patients with thoracolumbar fracture treated by surgery in our department were selected. The random number table method was used to divide into experimental group (*n* = 241) and control group (*n* = 241). Inclusion criteria are as follows: (1) patients with a thoracolumbar fracture who have undergone open surgery; (2) the operation was performed by the same doctor; and (3) age ≥18 years old. Exclusion criteria are as follows: (1) complicated with serious physical diseases; (2) those who have mental disorders and cannot cooperate with the investigation; (3) patients with original gastrointestinal organic diseases; and (4) there was a history of constipation before surgery.

### Research Methods

(1) The control group was given routine constipation care, specifically as follows: (1) Dietary guidance: patients were instructed to eat more coarse grains, vegetables, fruits and other cellulose-rich food, avoid eating spicy and stimulating food, easy to produce intestinal gas, give up smoking and alcohol, drinking water 2–3 L/d; (2) patients who had not defecated for 3 d were given oral laxatives, such as kaiserol and fruit guide tablets, as prescribed by the doctor; (3) manual assistance with defecation was used if all these treatments failed; and (4) patients should actively engage in functional exercises to prevent muscle atrophy and joint stiffness.

(2) The experimental group was given biofeedback therapy combined with cluster nursing based on the control group.

Biofeedback therapy: GAP-08A biofeedback therapy training system (Mida Medical Instrument Co., Ltd.) was used. Before training, explained the relevant precautions to the patients and helped them to know the defecation action and the way to coordinate muscles when defecating correctly. On the day of training, patients were instructed to empty urine and feces, all patients were placed in the lateral position, the proper amount of paraffin oil was used to smear the skin around the anus and biofeedback catheter with balloon, single-channel pressure measuring catheter and anal electrode were inserted into the anus and rectum of patients with a depth of about 9–10 cm, and the catheter was adjusted to the appropriate position according to the pressure curve. The anorectal pressure signal recorded and amplified by the computer can be displayed by the display, so that patients can identify whether their anorectal muscles were moving normally or not. At the same time, with the help of intuitive images and audio images of the biofeedback therapy system, the patients were instructed to understand the movement key points of abdominal exertion and anal relaxation during correct defecation. Constant training and feedback were conducted under real-time monitoring pressure, 40 min/time, 2–3 times a week, 10 treatments were given.

Cluster nursing: The orthopedic medical staff and relevant professionals of the rehabilitation department were convened to discuss together, and the clinically proven effective and recommended elements were incorporated into the clustered nursing plan. Standardized training was conducted for researchers, the concept of cluster nursing was introduced, and the evidence-based medicine basis and implementation approach of each element were understood. The researchers should analyze the current problems and shortcomings and make timely adjustments to the plan according to the specific situation of patients. The specific operations were as follows: (1) Health education: Constipation-related knowledge presentations were carried out to enable patients to understand its hazards and preventive measures. (2) The regular bowel habit was established, and patients were instructed to drink a glass of honey water or light salt water in the morning on the basis of a regular diet, and to instruct patients to chew and swallow slowly when eating. (3) Defecation-related muscle exercises: Patients were instructed to perform anus contraction exercises, abdominal muscle contraction exercises, abdominal deep breathing exercises, a total of 10–15 min. Patients with more than 6 thoracic fractures may have hypertension, coronary heart disease, and cerebrovascular accidents caused by excessive phytonerve reflexes during abdominal deep breathing exercises. Special attention needs to be paid to these patients clinically. (4) Rectal stimulation induces defecation: Before the operation, the nursing staff instructed the patient to empty the bladder and clean the hands in a six-step wash procedure. The patient took the supine position, and the nursing staff paid attention to the patient's umbilicus, gently and slowly massaged in a clockwise direction for 5–10 min. Then, nurses inserted the fingers into the patient's anus and rotated clockwise in the intestinal wall for 15–20 s, which could be repeated 3–5 times to induce reflex defecation in the rectum. If the above operations were ineffective or the bowel movement was incomplete, the nursing staff could use their fingers to assist the bowel movement. (5) Psychological support: Nurses gave full affirmation to patients, dealt with patients' negative emotions in time, encouraged patients to express their worries and distress, and provided psychological counseling to patients.

### Observation Index

All subjects were assessed 1 month after the intervention.

The basic data of patients were collected, such as gender, age, education level, marital status, and thoracolumbar injury severity score (TLISS).

The constipation score method was used to assess the severity of constipation, such as difficulty defecation, the defecation was incomplete, defecation time, and defecation times. The score of each item was grade 4 standard, with 0–3 points according to different degrees. Difficulty defecation: 0 point (none), 1 point (occasionally), 2 points (often), and 3 points (every time); the defecation was incomplete: 0 point (none), 1 point (occasionally), 2 points (obvious), and 3 points (very obvious); defecation time: 0 point (<5 min), 1 point (5–10 min), 2 points (11–16 min), and 3 points (>16 min); and defecation times: 0 point (1–2 days), 1 point (3 days), 2 points (4–5 days), and 3 points (>5 days). The higher the score, the more serious constipation was.

According to the Bristol stool scale, stool traits were divided into 7 types. Type 1: stool showed nut-shaped hard balls; Type 2: stool was lumpy but sausage-like; Type 3: stool was sausage-like with cracks on the surface; Type 4: stool surface was smooth and soft, but it looks like sausage; Type 5: soft lump stool; Type 6: pasty stool; and Type 7: watery stool. Type 1 corresponded to 1 point, type 2 corresponded to 2 points, and so on. The higher the score, the looser the stool was.

The short health questionnaire (SF-36) was used to evaluate patients' quality of life. The questionnaire consisted of 36 items, i.e., 8 dimensions: physical function, physical function, physical pain, general health, energy, social function, emotional function, psychological function, and additional health changes. The total score of each dimension was 100 points, and the final score was the average score of each dimension. The higher the score, the better the quality of life. The Cronbach's α coefficient of SF-36 was 0.871.

The satisfaction questionnaire made by our hospital was used to evaluate patients' satisfaction with nursing work, i.e., treatment effect, working ability, working attitude, and daily guidance. The total score was 100 points, and the survey results were divided into unsatisfied: <60 points, satisfied: 60–80 points, very satisfied: >80 points, total satisfaction = (very satisfied + satisfied)/total cases × 100%. The content validity index of the self-made satisfaction questionnaire was 0.90.

### Statistical Methods

SPSS22.0 software was used, and the measurement data were expressed as $\bar{x}$ ± s, and *t*-test was used for comparison. The counting data were expressed by %, and the χ^2^-test was used for comparison. *p* < 0.05, the difference was statistically significant.

## Results

### Baseline Data of Two Groups

The baseline data of the two groups were balanced and comparable (*p* > 0.05; Table 1).

**Table 1:** Baseline data of two groups (*n*, %, $\bar{x}$ ± s).

**Group**		**Control group (*n* = 241)**	**Experimental group (*n* = 241)**	***χ^2^/t-*value**	***P*-value**
Gender	Male	136 (56.43%)	129 (53.53%)	0.411	0.522
	Female	105 (43.57%)	112 (46.47%)		
Age (years)		49.31 ± 8.62	48.64 ± 8.17	0.875	0.381
Education level	Junior high school and below	87 (36.10%)	84 (34.85%)	0.520	0.771
	Senior high school	93 (38.59%)	89 (36.93%)		
	College degree or above	61 (25.31%)	68 (28.22%)		
Marital status	Married	169 (70.12%)	166 (68.88%)	0.585	0.746
	Be unmarried	42 (17.43%)	48 (19.92%)		
	Divorced or widowed	30 (12.45%)	27 (11.20%)		
TLISS score (point)		6.46 ± 1.31	6.37 ± 1.24	0.774	0.439

### The Severity of Constipation in Two Groups

**Figure 1:**
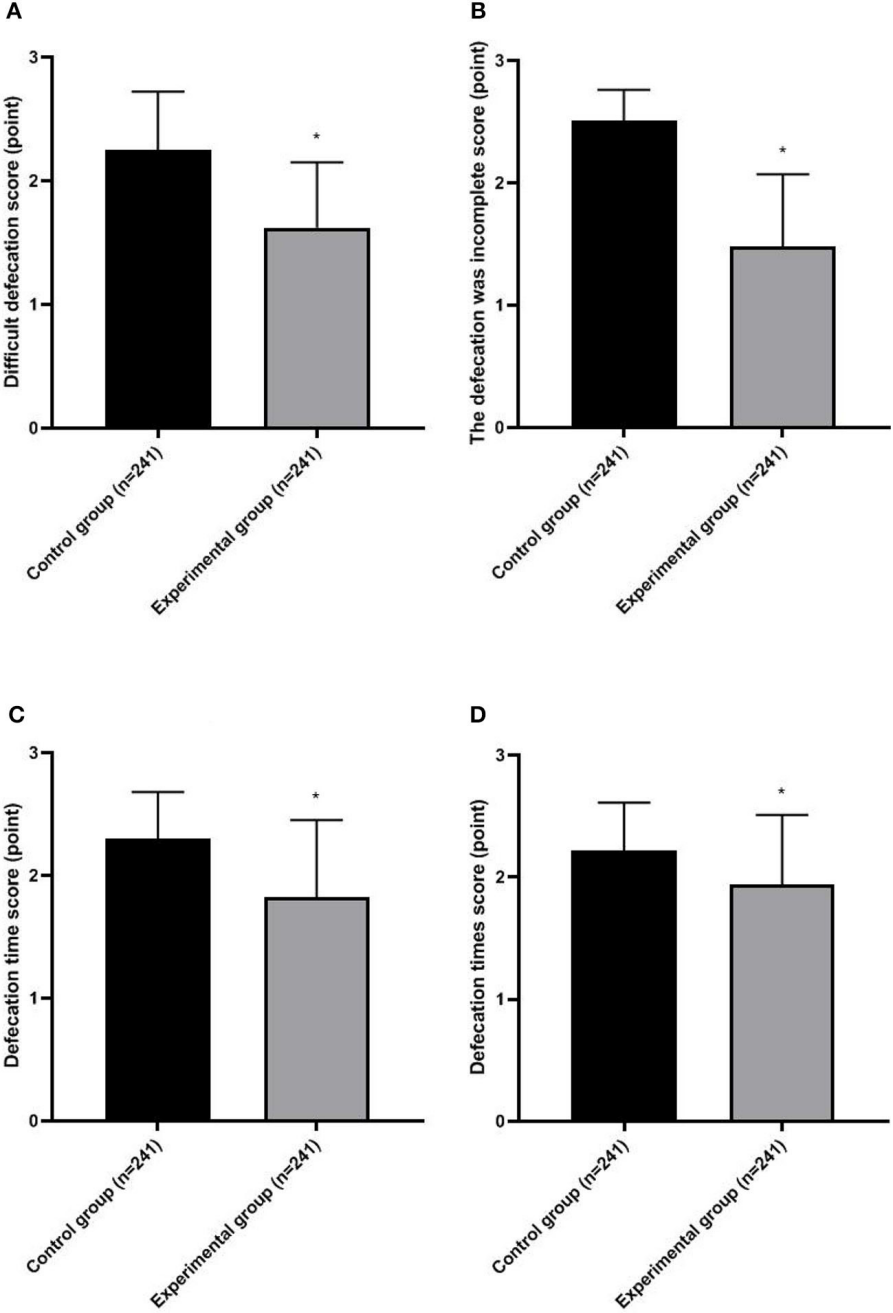
Severity of constipation in two groups. **(A)** Difficulty defecation score; **(B)** The defecation was incomplete score; **(C)** Defecation time score; **(D)** Defecation times score. Compared with the control group, *p < 0.05.

The constipation scores of the experimental group were lower than those of the control group (*p* < 0.05; Figure 1).

### Stool Traits of Two Groups

**Figure 2:**
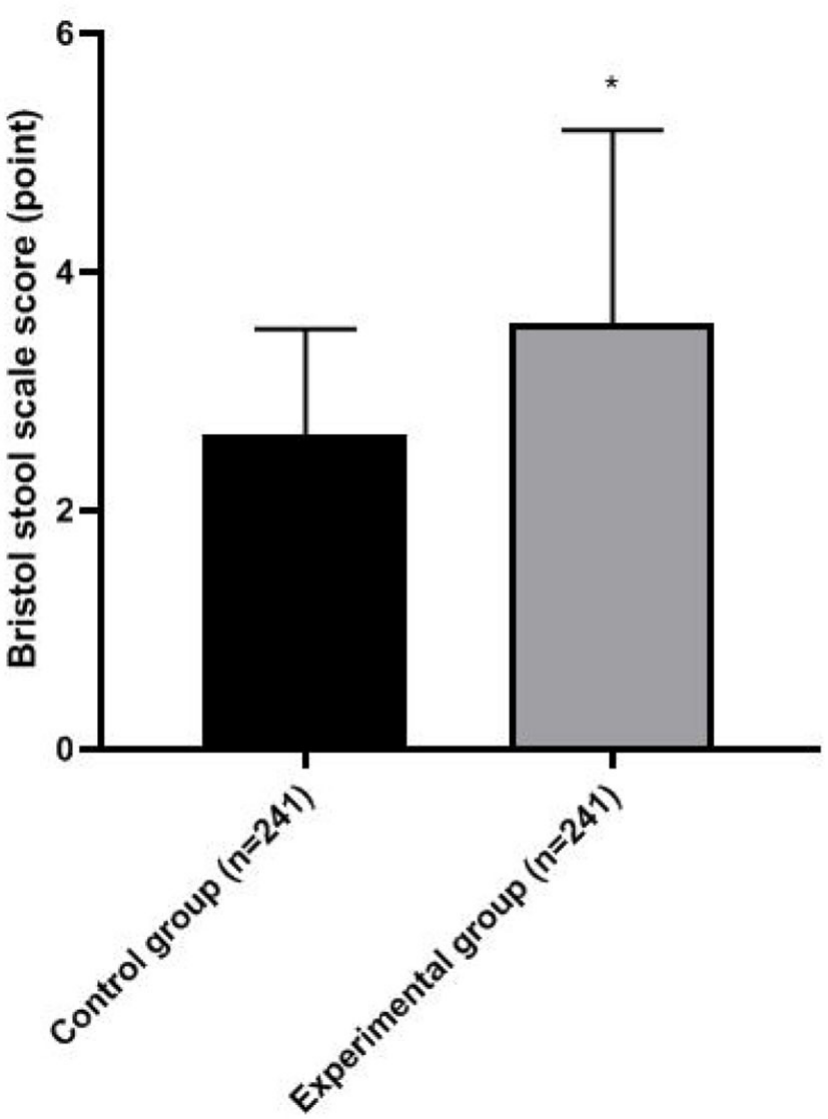
Stool traits of two groups. Compared with the control group, *p < 0.05.

The Bristol stool scale of the experimental group was higher than that of the control group (*p* < 0.05; Figure 2).

### Quality of Life of Two Groups

**Figure 3:**
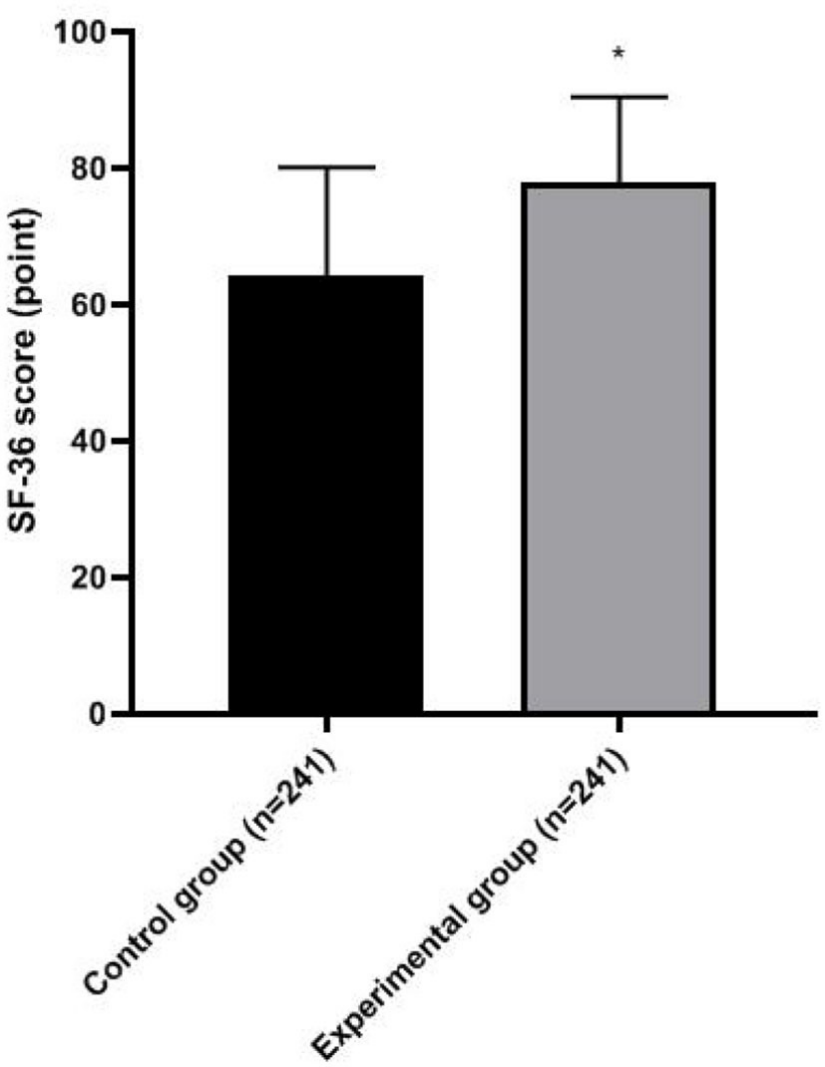
Quality of life of two groups. Compared with the control group, *p < 0.05.

The SF-36 score of the experimental group was higher than that of the control group (*p* < 0.05; Figure 3).

### Satisfaction of Two Groups

The satisfaction of the experimental group (90.46%) was higher than that of the control group (83.40%; *p* < 0.05; Table 2).

**Table 2:** Satisfaction of two groups (*n*, %).

**Group**	**Very satisfied**	**Satisfied**	**Dissatisfied**	**Total satisfaction**
Control group (*n* = 241)	117 (48.55%)	84 (39.25%)	40 (16.60%)	201 (83.40%)
Experimental group (*n* = 241)	145 (60.17%)	73 (30.29%)	23 (9.54%)	218 (90.46%)
*χ^2^-*value				5.277
*P*-value				0.022

## Discussion

Common causes of constipation in patients with a thoracolumbar fracture are as follows: abundant sympathetic nerves are distributed inside and outside the spinal canal, and injuries of thoracolumbar vertebrae can stimulate sympathetic nerves, leading to intestinal peristalsis dysfunction. After the fracture surgery, patients need to stay in bed for a long time, spend less time to get out of bed, and eat fewer fruits and vegetables, which leads to the decrease of systemic metabolism, weakened gastrointestinal activity, reduced colonic peristalsis, and excessive retention of colonic contents, which are closely related to constipation. Patients with the fracture are often accompanied by negative emotions, such as anxiety and irritability. Psychological stress may affect gastrointestinal function through the autonomic efferent nerve pathway (14–16). When constipation occurs in patients with thoracolumbar fractures, feces stay in the intestinal cavity for a long time, and wastes and poisons cannot be excreted in time, which will lead to abdominal distension, nausea, and loss of appetite and increase the pain of patients (17). Therefore, it is necessary to take effective clinical nursing measures to solve this problem.

Biofeedback therapy uses receptors to transform physiological activity information that the human body cannot be perceived into intuitive information that can be perceived, so that patients can accurately perceive their own physiological activities, and at the same time, guide patients to exercise, form a normal physiological feedback channel, so as to achieve the purpose of regulating their own physical functions (18). Skardoon's et al. team research has shown (19) that biofeedback therapy has a better therapeutic effect than drug therapy alone, and patients are well-tolerated by this therapy. About 88% of constipation patients can get an improvement of symptoms, and the benefit time of constipation patients can be as long as 44 months. Verma's et al. team reported (20) that biofeedback therapy can effectively improve constipation patients' defecation, reduce anal relaxation pressure during simulated defecation, increase the maximum rectal pressure during simulated defecation, and improve the coordination between intra-abdominal pressure and pelvic floor muscles during defecation, 62% of patients were satisfied with the intervention effect. At present, the routine clinical intervention methods have played a certain role in the constipation nursing of patients with thoracolumbar fracture, but the intervention content is relatively simple, lacking pertinence and comprehensiveness. Cluster nursing is a method to implement various nursing elements on the basis of evidence. According to the actual situation of hospitals and patients, making a series of personalized nursing measures, following the people-oriented principle, and the implementation of optimized nursing intervention can improve the comprehensive nursing effect to the greatest extent (21). This nursing model is patient-centered, with all-around intervention, all contents are in line with evidence-based medicine, and it has the advantages of practicality and integrity (22).

In this study, biofeedback therapy and cluster nursing were integrated and applied to patients with thoracolumbar fracture. The results showed that the constipation scores of the experimental group were lower than those of the control group, while the Bristol stool scale score, SF-36 score, and satisfaction degree of the experimental group were higher than those of the control group. This revealed that biofeedback therapy combined with cluster nursing has a good intervention effect in perioperative constipation of patients with thoracolumbar fracture, which can reduce the degree of constipation, improve stool traits, improve the quality of life, and improve the satisfaction of patients. Biofeedback therapy, with the help of intuitive vision and hearing provided by anorectal pressure measuring equipment, enables patients to visually perceive the muscle activity of the anus during defecation. After repeated training, they can understand the changes of figures or sounds corresponding to different defecation movements, learn the methods of relaxing pelvic floor muscles during defecation, adjust the coordination between abdominal and anorectal muscles, correct abnormal physiological activities, and help to improve the score of constipation symptoms of patients (23). We use the biofeedback system mediated by perfusion pressure sensor with a balloon catheter, the operation steps are simple, the pressure changes are sensitive, and the patient has well-tolerance. In cluster nursing, health education can effectively improve the bad living habits of patients and reduce constipation by adjusting diet and exercising properly. Drinking honey water and light salt water in the morning can lubricate the intestinal tract, replenish the body's water, and stimulate intestinal peristalsis, which help to improve defecation function. When eating, eating slowly can prevent patients from swallowing more air, thus reducing abdominal distension and achieving the purpose of defecation. The exercise of defecation-related muscles can strengthen the strength of patients' abdominal and pelvic muscles, while the anal contraction training and abdominal muscle training contraction can increase abdominal pressure, strengthen the contraction of abdominal and anal muscles, thus promoting rectal movement and increasing defecation feeling. Abdominal deep breathing training can massage and pull the internal organs, strengthen gastrointestinal peristalsis, and reduce constipation in patients. Abdominal massage is a kind of mechanical stimulation to the gastrointestinal tract, and signals are transmitted to the brain through the tactile sensation and pressure receptors of skin, and the sympathetic nervous system is reflexively excited, which promotes the downward movement of the contents of descending colon, thereby enhancing the body's metabolism and keeping the digestive system in a good balance. When the finger is inserted into the rectum and rotated clockwise in the intestinal wall, the signal of finger stimulation is equivalent to that of stool to the rectal wall, which is beneficial to stimulate the intestinal tract to empty the stool (24). In addition, the brain-intestine axis is a biochemical signal that constantly adjusts the internal and external environment of the body, and mental factors are closely related to intestinal function (25). Therefore, clustered nursing is a positive effect on reducing the incidence of constipation by giving full recognition to patients with thoracolumbar fracture, building self-confidence, and reducing unhealthy psychology. The combined application of biofeedback therapy and cluster nursing can Gather the strengths and complement the weaknesses, which has a better intervention effect on constipation of patients with thoracolumbar fracture.

## Conclusion

Through this study, it can be known that biofeedback therapy combined with cluster nursing has a good intervention effect in perioperative constipation of patients with thoracolumbar fracture, which can reduce the degree of constipation, improve stool traits, improve the quality of life, and improve the satisfaction of patients. However, this study is a single-center study with a short observation time, and the results of this study may be interfered by many factors. Further multi-center studies and extended study time are needed to clarify the conclusion.

## Data Availability Statement

The original contributions presented in the study are included in the article/supplementary material, further inquiries can be directed to the corresponding author/s.

## Ethics Statement

The studies involving human participants were reviewed and approved by the Ethics Committee of Suining Central Hospital. The patients/participants provided their written informed consent to participate in this study.

## Author Contributions

JL conducts research design and writes papers. NX evaluates test results and conducts data statistics. LY guides the entire research. All authors contributed to this research, contributed to the article, and approved the submitted version.

## Conflict of Interest

The authors declare that the research was conducted in the absence of any commercial or financial relationships that could be construed as a potential conflict of interest.

## Publisher's Note

All claims expressed in this article are solely those of the authors and do not necessarily represent those of their affiliated organizations, or those of the publisher, the editors and the reviewers. Any product that may be evaluated in this article, or claim that may be made by its manufacturer, is not guaranteed or endorsed by the publisher.
